# Hydrogen Production from Barley Straw and Miscanthus by the Hyperthermophilic Bacterium, *Cadicellulosirupter bescii*

**DOI:** 10.4014/jmb.2305.05022

**Published:** 2023-06-19

**Authors:** Minseok Cha, Jun-Ha Kim, Hyo-Jin Choi, Soo Bin Nho, Soo-Yeon Kim, Young-Lok Cha, Hyoungwoon Song, Won-Heong Lee, Sun-Ki Kim, Soo-Jung Kim

**Affiliations:** 1 Research Center for Biological Cybernetics, Chonnam National University, Gwangju 61186, Republic of Korea; 2 Department of Integrative Food, Bioscience and Biotechnology, Chonnam National University, Gwangju 61186, Republic of Korea; 3 Department of Food Science and Biotechnology, Chung-Ang University, Gyeonggi 17546, Republic of Korea; 4 Bioenergy Crop Research Institute, National Institute of Crop Science, Rural Development Administration, Muan 58545, Republic of Korea; 5 Institute for Advanced Engineering, Gyeonggi 17180, Republic of Korea

**Keywords:** Biohydrogen, *Caldicellulosirupter bescii*, Barley straw, Miscanthus

## Abstract

This work aimed to evaluate the feasibility of biohydrogen production from Barley Straw and Miscanthus. The primary obstacle in plant biomass decomposition is the recalcitrance of the biomass itself. Plant cell walls consist of cellulose, hemicellulose, and lignin, which make the plant robust to decomposition. However, the hyperthermophilic bacterium, *Caldicellulosiruptor bescii*, can efficiently utilize lignocellulosic feedstocks (Barley Straw and Miscanthus) for energy production, and *C. bescii* can now be metabolically engineered or isolated to produce more hydrogen and other biochemicals. In the present study, two strains, *C. bescii* JWCB001 (wild-type) and JWCB018 (*ΔpyrFA Δldh ΔcbeI*), were tested for their ability to increase hydrogen production from Barley Straw and Miscanthus. The JWCB018 resulted in a redirection of carbon and electron (carried by NADH) flow from lactate production to acetate and hydrogen production. JWCB018 produced ~54% and 63% more acetate and hydrogen from Barley Straw, respectively than its wild-type counterpart, JWCB001. Also, 25% more hydrogen from Miscanthus was obtained by the JWCB018 strain with 33% more acetate relative to JWCB001. It was supported that the engineered *C. bescii*, such as the JWCB018, can be a parental strain to get more hydrogen and other biochemicals from various biomass.

## Introduction

**Fig. 1:**
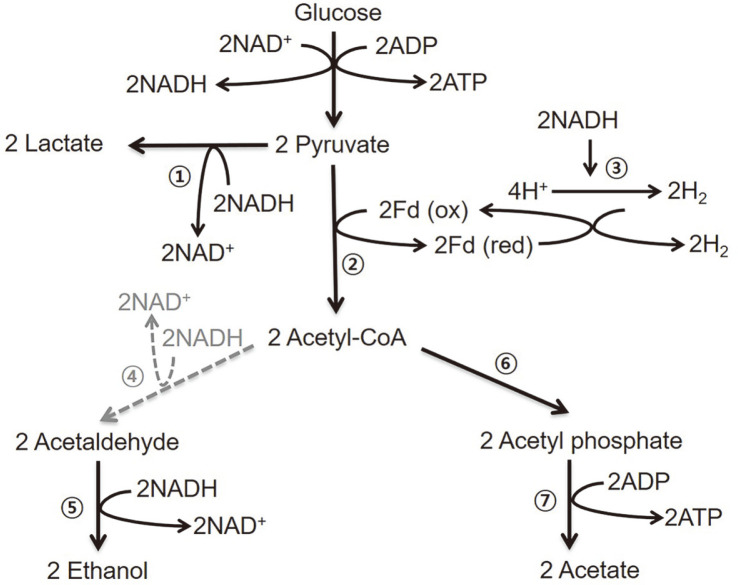
Proposed metabolic pathways in *Caldicellulosiruptor bescii*. (1) L-Lactate dehydrogenase; (2) Pyruvateferredoxin oxidoreductase; (3) Bifurcating (reduced ferredoxin:NADH-dependent) hydrogenase; (4) Aldehyde dehydrogenase; (5) Alcohol dehydrogenase; (6) Phosphotransacetylase; (7) Acetate kinase. The light gray dotted arrow represents potential heterologous pathways that do not exist in *C. bescii*, but could be genetically engineered using the parental strain, JWCB018.

In the near future, we will be required to contend with serious environmental problems such as the greenhouse effect, global climate change, and fine dust caused by the widespread use of fossil fuels. Fuel production from plant biomass is a potential remedy for many of these problems. However, plants have evolved to resist decomposition by microorganisms, and plant cell walls consist of cellulose, hemicellulose, and lignin, which can make the plant recalcitrant [1-4]. *Caldicellulosiruptor bescii* is able to utilize lignocellulosic feedstocks efficiently and is also the most thermophilic/cellulolytic bacterium, with an optimal growth temperature of 70°C ~ 80°C [5-7]. *C. bescii* also can utilize C5 and C6 sugars released from plant biomass. The carbohydrates are oxidized in the Embden–Meyerhof–Parnas pathway producing acetate, lactate, CO_2_, and hydrogen as major fermentative end products (Fig. 1) [7-9]. In this pathway, pyruvate is the major metabolic branch point during fermentation, routing carbon to lactate or acetyl-CoA and electrons carried by nicotinamide adenine dinucleotide (NADH) to lactate or H_2_. At this point, the acetate production pathway is essential for H_2_ production, permitting the re-oxidation of NADH and ferredoxin, which can be simultaneously oxidized by a bifurcating hydrogenase [9]. Hydrogen is currently the most actively studied biofuel of these fermentative end products. There are already many bacterial strains that produce high yields of hydrogen, especially *Thermoanaerobacter tengcongensis* (~ 4.0 mol/ mol glucose, [10]), *Thermotoga maritima* (~ 4.0 mol/mol glucose, [11]), *Thermococcus kodakaransis* (~ 3.3 mol/mol glucose, [12]), etc. Although *C. bescii* has special advantages for the conversion of plant biomass to fuels and chemicals, this strain also possesses a strong Restriction-Modification (R-M) system, which fundamentally limits DNA transformation and, in turn, limits the practicality of using this strain for commercial purposes at scale [13]. To overcome the R-M system in *C. bescii*, Chung, *et al*. constructed a mutant strain, JWCB018, by deleting the endonuclease encoding gene (*cbeI*), rendering this strain more easily engineered [13]. *C. bescii* is an attractive platform for metabolic engineering for maximal H_2_ production from various plant biomass (especially Barley Straw and Miscanthous), partly because it could significantly reduce processing costs compared to current fuel production method from biomass.

Here, we provide evidence of highly efficient H_2_ production by *C. bescii* by comparing a *C. bescii* wild-type, JWCB001, and a mutant strain, JWCB018 (*ΔpyrFA* *ΔldhΔ cbeI*). It will be feasible to utilize *C. bescii* to efficiently produce a significant amount of H_2_ from plant biomass by reprogramming the bioenergetic pathways of *C. bescii*, such as altering the acetate production towards H_2_ production.

## Materials and Methods

### Procurement and Validation of *C. bescii* Strains

A mutant strain (*ΔpyrFA* *ΔldhΔ* *ΔcbeI*), JWCB018, was constructed by Chung, *et al*. [13], and it spontaneously became a double mutant (*ΔcbeI* *Δldh*) by an active transposon on the lactate dehydrogenase-encoding gene (*ldh*)[14]. The *ΔcbeI* deletion strain was constructed based on JWCB005, which was a *pyrFA*-deleted strain of *C. bescii* [15]. Cha *et al*. isolated and purified the double mutant [14]. The two *C. bescii* strains, a wild-type (JWCB001) and a mutant (*ΔpyrFA* *ΔcbeI* *Δldh*, JWCB018), were obtained from Dr. Janet Westpheling’s Lab at the University of Georgia, USA (Table 1). The strains were stored in 10% DMSO (dimethyl sulfoxide) at -80°C immediately upon receipt. Deletions were confirmed by Polymerase Chain Reactions (PCRs) with specific sets of primers. To confirm a *cbeI* deletion, an external PCR and an internal PCR of *cbeI* were performed with primer sets, MC011/MC012 and MC011/MC013, respectively (Table 1). To confirm *ldh* interruption for modification by transposon insertion, an external PCR of *ldh* was performed with a primer set, MC014/MC015 (Table 1). All PCR products were evaluated by gel electrophoresis and visualized on 0.8% agarose gels.

### Growth Media and Conditions

*C. bescii* JWCB001 and JWCB018 (Table 1) were anaerobically grown in low osmolarity defined (LOD) medium [16] containing 40 μM MOPS with a final pH of 7.0, supplemented with cellobiose (1.0% (wt/vol); catalog no. 01407; Chem-Impex, USA) or maltose (1.0% (wt/vol); catalog no. 70090-0401; Junsel, Korea) as the carbon source, unless otherwise noted. The liquid cultures for the two strains were grown from a 2% inoculum, then incubated at 75°C in anaerobic culture bottles degassed with 7 cycles of vacuum and argon. An auxotrophic mutant, JWCB018, was grown in LOD medium supplemented with 40 μM uracil.

### Biomass Pretreatment

*Hordeum vulgare* (Barley straw) and *Miscanthus* (Miscanthus) were obtained from Jeonnam, Korea in 2021. The air-dried biomass was chopped to a length of 5 cm using a tub grinder (Tomotech Ltd., Korea). The chopped biomass was then ground using a 20 hp hammer mill (Sunbrand Industrial Inc., Korea) with 3.0 mm screens, dried at 60°C for 24 h, and then stored in desiccators. The chemical analysis indicated that the biomass mainly consisted of 35-43% cellulose, 22-25% hemicellulose, 18-22% lignin, and 3-7% ash.

Biomass was pretreated in an 800 ml pressure vessel equipped with a temperature and pressure sensor. The mixture of biomass and alkali catalyzed organic solvent (1:9, 300 ml working volume) was then loaded into the vessel. The alkali catalyzed organic solvent contained 12.2% sodium hydroxide per dried biomass volume in 57.4% ethanol. The vessel was then heated to 163°C for 60 min. Nitrogen gas was additionally loaded to 6 MPa in the vessel for explosion before the pretreated biomass was collected into a separator via pressure and temperature differences. The solid hydrolysate was obtained using a Buchner funnel with a 10 μm nylon filter and neutralized with tap water.

### Determination of Cell Growth, Carbon Sources, and By-Products during Fermentation

The strains were grown in stoppered 125-ml serum bottles containing 50 ml LOD medium supplemented with different carbon sources. Medium for the JWCB018 was supplemented with 1 mM uracil. Duplicate bottles were inoculated with a fresh 2% (vol/vol) inoculum and incubated at 75°C with shaking at 150 rpm. Optical cell density was monitored using a Biomate5 UV-visible spectrophotometer (Thermo Fisher, USA) measuring absorbance at 680 nm. The *C. bescii* wild-type and mutant strain were incubated in the same culture conditions, supplemented with 10 g/l (wt/vol) cellobiose, 20 g/l (wt/vol) Avicel (catalog no. 11365; Sigma-Aldrich, USA), 10 g/l (wt/vol) pretreated Barley Straw and 10 g/l (wt/vol) pretreated Miscanthus as a single carbon source, respectively.

Fermentative products, cellobiose, acetate, and lactate were analyzed on an Agilent Technologies 1200 Series HPLC system (Agilent Technologies, USA). Metabolites were separated on a Rezex ROA-Organic Acid H+ (8%) column (Phenomenex, USA) under isocratic temperature (60°C). Five mM H_2_SO_4_ was used as a mobile phase at a flow rate of 0.6 ml/min, and then the samples were passed through a refractive index detector (Agilent 1200 Infinity Refractive Index Detector). Identification of separated chemicals was compared to retention times with standards, and total peak areas were integrated and compared with peak areas and retention times of known standards for each compound of interest.

### Determination of H_2_ Production

The culture bottles were cooled to room temperature after 48 h of incubation at 75°C, and H_2_ was separated on an Agilent Technologies 8890 GC system (Agilent), equipped with a thermal conductivity detector (TCD) at 200°C and N_2_ reference flow, using the Agilent J&W CP-Molsieve 5Å CP 7535 column (Agilent) at 30°C. The hydrogen peak was isolated by comparing retention times with H_2_ standards. To measure H_2_ concentration produced, total peak areas were integrated and compared to peak areas and retention times of known H_2_ standards.

## Results

### Confirmation *cbeI* Deletion and Interruption of *ldh* Expression on the *C. bescii* Chromosome

**Fig. 2:**
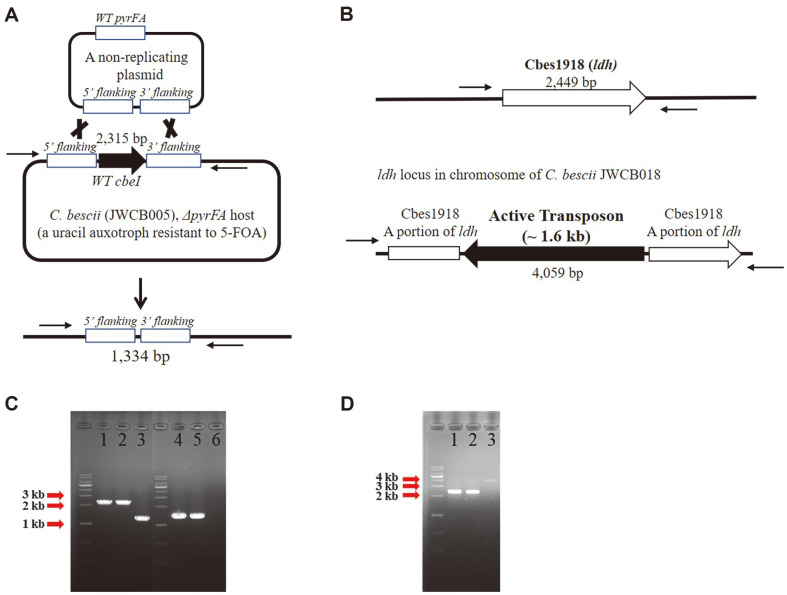
Confirmation of *cbeI* (Cbes2438) deletion and IS element insertion within *ldh* (Cbes1819) open reading frame in *C. bescii*. (**A**) Construction of *cbeI* deletion, (**B**) Simplified diagrams of *ldh* loci in chromosomes of *C. bescii* strains, (**C**) Agarose gels showing amplified PCR products from the outside (lane 1and 2 wild-type of *cbeI* locus; lane 3, *cbeI* deletion; expected bands: wild-type *cbeI* locus – 2.4 kb; *cbeI* deletion – 1.4 kb) and inside (lane 4 and 5, wild-type of *cbeI* locus; lane 6, *cbeI* deletion; expected bands: wild-type *cbeI* locus – 1.3 kb; *cbeI* deletion – no band), (**D**) Agarose gels showing amplified PCR products from the *ldh* locus (lane 1 and 2, wild-type; lane 3, IS interruption on ldh; expected bands: wild-type *cbeI* locus – 2.4 kb; *cbeI* deletion – 4.1 kb).

Two *C. bescii* strains, JWCB001 and JWCB018, were obtained from Dr. Janet Westpheling’s Lab at the University of Georgia, USA (Table 1). Chung *et al*. successfully constructed a deletion of *cbeI* (Cbes2438) based on JWCB005 (*ΔpyrFA* [13, 15]), using the targeted marker replacement strategy described in Fig. 2A [13]. This modification overcomes the R-M system in *C. bescii* [13]. To confirm the *cbeI* deletion in the obtained JWCB018, the region of the *cbeI* locus was amplified by PCRs, using two sets of primers directed outside (external PCR) and inside (internal PCR) the *cbeI* region of the chromosome (Fig. 2C). External primers were MC011/MC013; internal primers were MC011/MC013 (Table 1). The wild-type (JWCB001) strain gave the expected 2.4 kb (Fig. 2C, lane 1, 2) and 1.4 kb (Fig. 2C, lane 4, 5). bands, while PCR of the JWCB018 construct resulted in the predicted smaller 1.3 kb bands (Fig. 2D, lane 1, 2) and absence of bands (Fig. 2D, lane 3). The insertion of the *ldh* that interrupts *ldh* expression was identified and isolated by Cha *et al*. (Fig. 2B, [14]). We independently confirmed the insertion by PCR using primer set MC014/MC015 (Table 1). The PCR products from JWCB001 genomic DNA show 2.4 kb bands (Fig. 2D, lane 1, 2) and a PCR run on JWCB018 genomic DNA shows a 4.1 kb band (Fig. 2D, lane 3), as expected.

### Comparison of Growth and Hydrogen Production in the *C. bescii* Strains

**Fig. 3:**
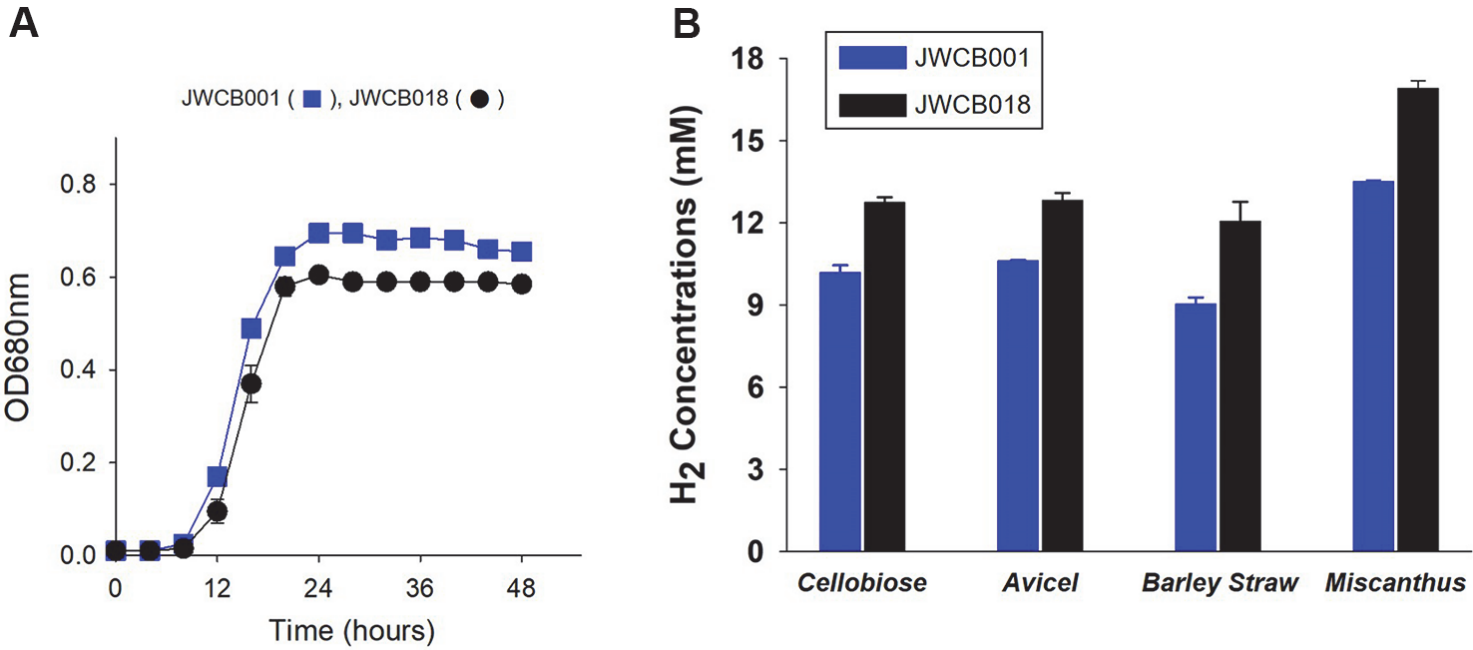
Comparison of growth (OD_680 nm_) and hydrogen production for the wild-type, JWCB001, and the mutant, JWCB018, strains. (**A**) Growth of *C. bescii* strains on 1% cellobiose as a sole carbon source; blue square, JWCB001; black circle, JWCB018 (*ΔpyrFA* *Δldh* *ΔcbeI*). (**B**) Hydrogen production on different carbon sources by each strain at the end of incubation (48 h); blue bars, JWCB001; black bars, JWCB018. Error bars based on two biologically independent experiments.

Growth rates of *C. bescii* JWCB001 and JWCB018 were compared when strains were cultured in LOD media [16] supplemented with 1.0% cellobiose (Fig. 3A). The growth rate of JWCB018 in the exponential phase was indistinguishable from that of the wild-type, JWCB001, although the final growth of the mutant strain, JWCB018, revealed a ~ 10% lower cell density than the wild-type (Fig. 3A).

For measurement and comparison of hydrogen production on different carbon sources, both *C. bescii* strains, JWCB001 and JWCB018, were grown on LOD medium [16] supplemented with 1.0% cellobiose (wt/vol), 2.0%Avicel (wt/vol), 1.0% Barley Straw (wt/vol) and 1.0% Miscanthus (wt/vol) as a carbon source, respectively (Fig. 3B). In the JWCB018, more NADHs was available for hydrogen production because the lactate production pathway was removed by an active transposon (Figs. 2B and 2D, [14]). The more available NADHs carry more electrons to the H_2_ production pathway, increasing H_2_ production in the cells. The mutant strain, JWCB018, produced 25% more H_2_ on 1% cellobiose and 21% more on 2% Avicel, compared to its wild-type counterpart, JWCB001 (Fig. 3B). Interestingly, the H_2_ production data indicated that JWCB018 produced 33% and 25% more H_2_ on 1.0% Barley Straw and 1.0% Miscanthus, respectively, than JWCB001 did (Fig. 3B), and significantly more H_2_ was produced on the 1% Miscanthus.

### Comparison of the Final Fermentation Products and Carbon Balances of *C. bescii* Wild-Type and Mutant Strains

**Fig. 4:**
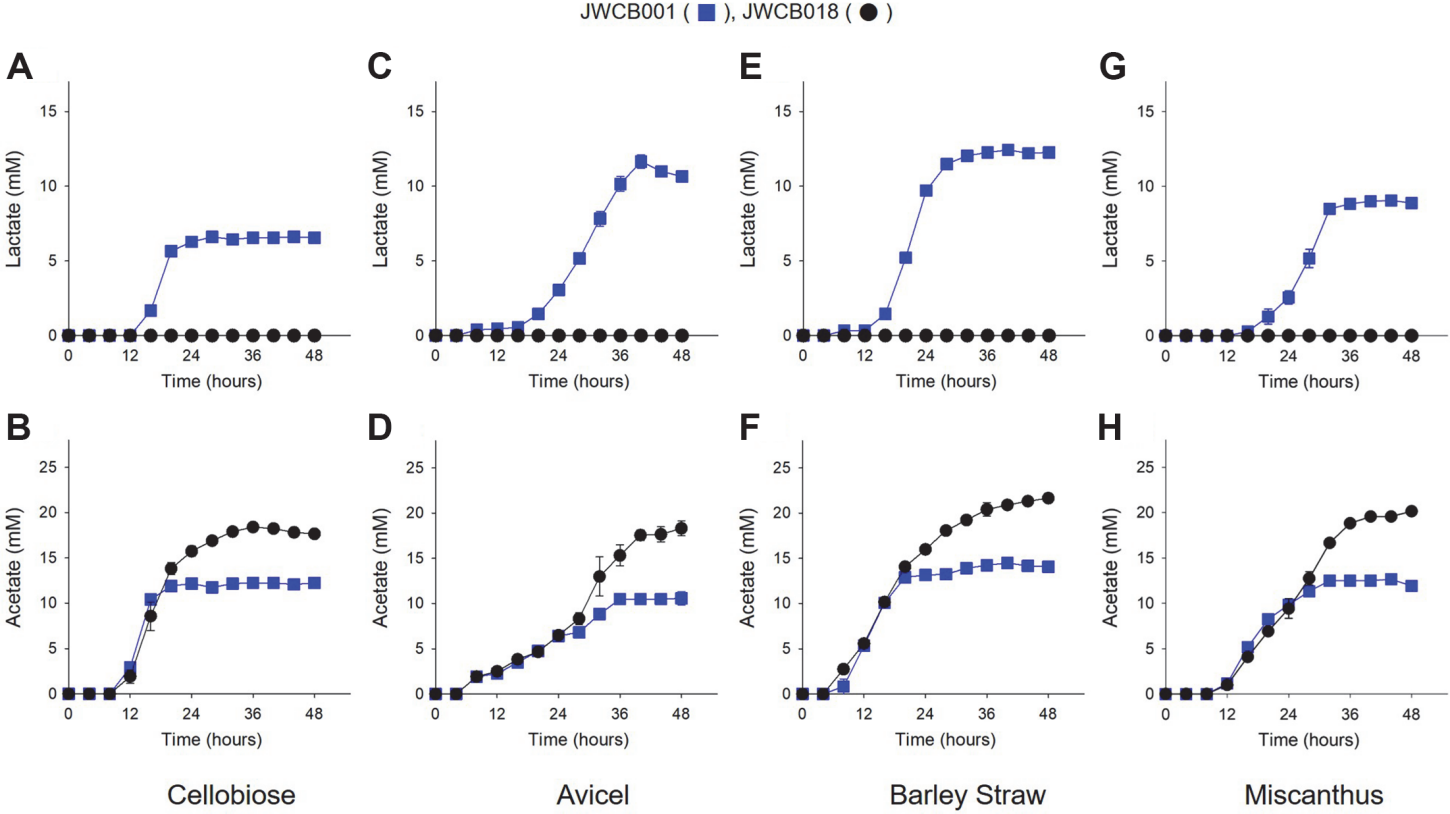
Comparing fermentative end products, lactate and acetate, of *C. bescii* strains, JWCB001 and JWCB018. Analysis of fermentation products lactate (**A, C, E**, and **G**) and acetate (**B, D, F**, and **H**), growing on 1% cellobiose (**A** and **B**), 2% Avicel (**C** and **D**), 1% Barley Straw (**E** and **F**), and 1% Miscanthus (**G** and **H**) at 75°C. Blue square, wild-type JWCB001; Black circle, JWCB018 (*ΔpyrFA* *Δldh* *ΔcbeI*). Error bars based on two biological replicates.

To compare the final fermentation products, *C. bescii* wild-type and mutant strains were grown in LOD medium [16] with 1% (wt/vol) cellobiose, 2% (wt/vol) Avicel, 1% (wt/vol) Barley Straw, or 1% (wt/vol) Miscanthus as a carbon source, respectively. The fermentation products were monitored by HPLC over the course of 48 h (Fig. 4), and final carbon utilization was calculated (Table 2). Product yield was calculated as product yield per mole cellobiose (mol/mol). The HPLC analysis showed that the *C. bescii* wild-type, JWCB001, produced lactate (6.5 mM from cellobiose, 10.5 mM from Avicel, 12.3 mM from Barley Straw, and 8.8 mM from Miscanthus) and acetate (12.2 mM from cellobiose, 10.6 from Avicel, 14.1 mM from Barley Straw, and 11.9 mM from Miscanthus) by the end of the time course (48 h, Fig. 4). The mutant strain (JWCB018, *ΔldhΔcbeI*) did not produce lactate over the same time frame. However, it produced more acetate (17.7 mM from cellobiose, 18.3 mM from Avicel, 21.6 mM from Barley Straw, and 20.2mM from Miscanthus) than wild-type by the end of the time course (48 h, Fig. 4). The JWCB018 (*ΔpyrFA* *Δldh* *ΔcbeI*) produced more acetate than wild-type, JWCB001, because its carbon flow to acetate was increased by the inactivation of *ldh* (Fig. 4).

The carbon mass balance for the end products of growth on 1% cellobiose was calculated at the end of the time course (48 h, Table 2). The wild-type yielded 1.2 mol/mol lactate, 2.2 mol/mol acetate, and 1.8 mol/mol hydrogen, with 94% overall carbon recovery (Table 2). The mutant strain, JWCB018 (*ΔpyrFA* *Δldh* *ΔcbeI*), did not produce lactate at all and yielded 2.9 mol/mol acetate and 2.1 mol/mol hydrogen, with 90% carbon recovery overall (Table 2).

## Discussion

Based on the genome sequence of *C. bescii*, there is only one predicted lactate dehydrogenase gene (Cbes1918). To confirm this, both JWCB001 and JWCB018 were grown on different carbon sources. The JWCB018 mutant (Cbes1918 expression interrupted by an active transposon (Figs. 2B and 2D)) produced no detectable lactate. On the other hand, the wild-type, JWCB001, demonstrated lactate productions of 6.5 mM on cellobiose, 10.6 mM on Avicel, 12.3 Mm on Barley Straw, and 8.8 mM on Miscanthus (Fig. 4). Instead of lactate, the JWCB018 mutant strain produced much more acetate and hydrogen due to increased carbon and electron (carried by NADH) flux to acetate and hydrogen, respectively. To compare the production of acetate and hydrogen, *C. bescii* JWCB001 and JWCB018 were grown in LOD medium [16] with soluble cellobiose or real-world biomass (Barley Straw and Miscanthus) as a carbon source. JWCB018 produced 54% more acetate and 25% more hydrogen than JWCB001 when both strains were grown on 1% cellobiose for 48 h (Figs. 3 and 4). When the strains, JWCB001 and JWCB018, were grown on 1% Barley Straw as the sole carbon source, they showed a very similar profile to that of cellobiose. The *Δldh* strain, JWCB018, showed 54% and 63% more acetate production and 33% and 25% hydrogen production on Barley Straw and Miscanthus, respectively, than JWCB001 (Figs. 3 and 4). The JWCB018 produced more acetate and hydrogen on 1% Barley Straw and Miscanthus than on 1% cellobiose because the plant biomass (Barley Straw and Miscanthus) consists of cellulose, hemicellulose, and lignin, which can all be effectively decomposed by *C. bescii* [5-7].

In this study, we provide evidence for the effective production of biohydrogen from the real-world biomass by *C. bescii* strains. Since the endonuclease-encoding gene (*cbeI*) in *C. bescii* mutant strain was deleted, no R-M system exists in the cells. This permits easy metabolic engineering of the strain to optimize its hydrogen production from the real-world biomass. Other effective modifications could include eliminating the acetate production by deletion of *ak* and *pta* genes coding for key enzymes in the acetate biosynthetic pathway, or heterogeneous expression of strong hydrogenases from other thermophiles. Alternatively, a strong promotor could be inserted to amplify H_2_ production. Due to its versatility, the *C. bescii* mutant strain, JWCB018, lends itself well to rational strain engineering and can serve as a parent strain for production of biohydrogen at scale from lignocellulosic feedstocks.

## Figures and Tables

**Table 1:** Strains and primers used in this study.

Strains	Strain and genotype/phenotype	Sources
JWCB001	*C. bescii* DSM6725	DSMZ^1^
JWCB018	*C. bescii* *ΔpyrFA* *Δldh* *ΔcbeI* / (ura^-^/5-FOA^R^)	[13, 16]
Primers	Sequences (5’ to 3’)	
MC011	ATC ATC GTA CGT TAT CAT CCA CAG GTG	This study
MC012	TTC AAG AGC CTG GTG TAT CTC CTG C	This study
MC013	CAA CGT GGT GAT GTA AGA GAT ATG TTA GC	This study
MC014	ATC TTG CCA CGT ACA ATC TCT CCT TCA G	This study
MC015	TCT CTG ATA ATA TGG CCC AGG AGA TTA TTC TTC	This study

^1^German collection of microorganisms and cell cultures.

**Table 2:** Values for carbon balance of the end products at 48 h fermentation.

Strains	Concentrations of residual compounds (mM)							
	Initial Cellobiose	Final Cellobiose	Glucose	Cellobiose	Lactate	Acetate	Hydrogen	Carbon Recovery (%)
JWCB001	30.9 ± 0.01	12.0 ± 0.22	26.6 ± 0.56	5.6 ± 0.07	6.6 ± 0.11	12.2 ± 0.08	10.2 ± 0.27	94
JWCB018	30.8 ± 0.06	19.6 ± 0.19	10.6 ± 0.47	6.0 ± 0.01	0.0	17.7 ± 0.17	12.7 ± 0.19	90
